# Reading the Evolutionary History of the Woolly Mammoth in Its Mitochondrial Genome

**DOI:** 10.1371/journal.pbio.0040074

**Published:** 2006-02-07

**Authors:** Liza Gross

The woolly mammoth *(Mammuthus primigenius)* disappeared along with the last ice age over 10,000 years ago, yet scientists can glean insight into its evolutionary history by studying its frozen remains. Three recent independent studies applied different methods to isolate and sequence multiple DNA fragments from mammoth specimens. Two teams, reporting in December 2005, used mammoth bones extricated from the frozen permafrost of Siberia. One team, reporting in *Nature*, analyzed the mitochondrial DNA (mtDNA)—genetic material found in the cell's energy-producing organelles and transmitted only through the mother—of a 12,000-year-old specimen and concluded that the mammoth's closest living relative is the Asian elephant. The other team, reporting in *Science*, analyzed a portion of nuclear and mitochondrial DNA from a 28,000-year-old specimen, using a new method that increases the quantity of DNA fragments that can be extracted and sequenced from ancient specimens.

Ancient DNA studies have traditionally focused on mtDNA, but these investigations have relied on mostly short fragments of mtDNA. In the third and most recent study, Evgeny Rogaev, Yuri Moliaka, and their Russian colleagues sequenced the complete mitochondrial genome of a 33,000-year-old mammoth specimen. (Radiocarbon dating indicated that the mammoth lived 32,850 years ago, plus or minus 900 years.) This is the oldest sequenced mitochondrial genome and—at 16,842 base pairs—the longest stretch of sequenced DNA from an extinct Pleistocene species. This analysis, like the *Nature* study, points to the Asian elephant as the mammoth's closest relative. Interestingly, the Rogaev et al. study also suggests that genetic diversity among mammoths living in northeastern Siberia during the late Pleistocene was low.

Having complete mitochondrial mammoth genomes will greatly aid researchers' attempts to refine the elephant family tree and explore the genetic variation within this species. Notably, artifact mutations related to chemical modification of ancient DNA commonly contribute to errors in sequence, confounding estimates of diversity between species or individuals. The long sequences used in the Rogaev et al. study largely avoided this problem.

The ancient specimen, a right hind leg, was so well preserved that Rogaev et al. could extract DNA from mitochondria-rich muscle tissue, rather than bone. Two different labs generated multiple mtDNA products using PCR (polymerase chain reaction, the standard method for making millions of copies of DNA for sequencing) and reconstructed the mammoth genome sequence. The mtDNA sequence produced in the two labs appeared to be identical. So much DNA was retrieved that the authors needed only one round of PCR amplification, reducing the likelihood of sequence errors associated with the repeated amplification rounds needed for short DNA fragments, a longstanding problem with ancient DNA.

To determine the prehistoric pachyderm's evolutionary position relative to its Asian *(Elephas maximus)* and African *(Loxodonta Africana)* cousins, the authors also sequenced the complete mtDNA genomes of the two modern elephants from Africa and Asia. Rogaev et al. used the mammoth and elephant genomes, along with previously published mtDNA Asian and African elephant genomes, to construct an elephant evolutionary tree. They used two other placental mammals, the dugong and hyrax, to calibrate the tree, and show that the mammoth and Asian elephant are more closely related to each other than either is to the African elephant. Additional analysis using nuclear DNA and more closely related mammals could help refine the tree.

The authors also compared their sequence with other available mammoth sequences (including the recently published *Nature* sequence) representing populations from different locations and time periods. This analysis revealed the relatively low genetic diversity in the maternal lineage of mammoths in northeastern Siberia through the roughly 20,000-year period of the late Pleistocene. The population structure of mammoths living at that time appears less complex than that seen in the genomes of Asian and African elephants.

The mammoth and Asian elephant may have diverged around 4 million years ago, splitting soon after their lineage diverged from the African elephant. The authors point out that, given the inherent differences in mutation rates and mode of inheritance between nuclear and mitochondrial DNA, studies focusing on one or the other may produce different dates for the last common ancestor.

But these results, together with the *Nature* and *Science* studies, demonstrate the tractability of recovering large amounts of high-quality DNA from ancient extinct Pleistocene animals. Rogaev et al. found that relatively large, nondegraded DNA fragments may be extracted from animals preserved in the permafrost for many thousands of years, allowing complete genes (at least from mitochondrial genome) of extinct animals to be cloned. As scientists continue to mine the DNA-rich permafrost, they will be able to weave together the missing pieces of the evolutionary story of a long-lost, fabled creature and its modern relatives, sequence by sequence. To learn more about ancient DNA research, see the related primer (DOI: 10.1371/journal.pbio.0040078).

## 

**Figure:**
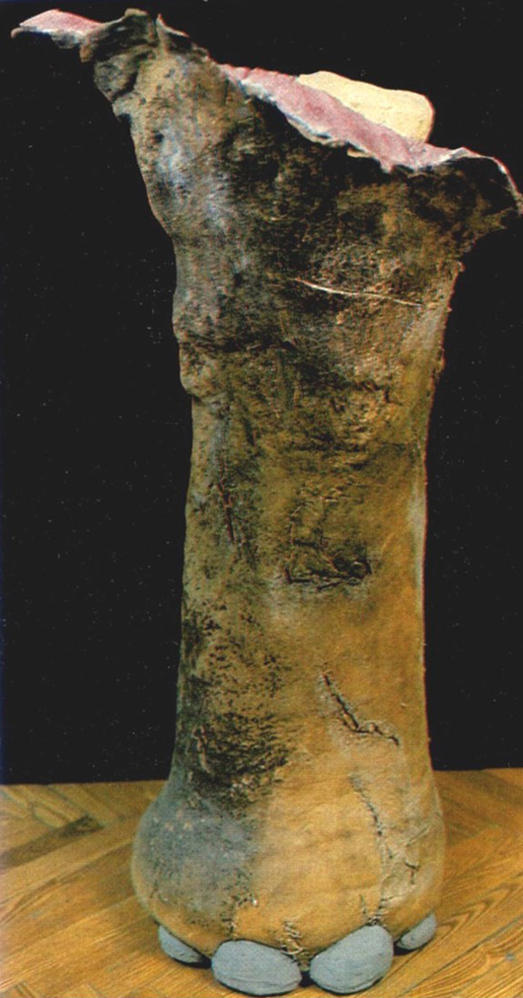
The restored right back leg of a 33,000-year-old woolly mammoth found in northeastern Siberia.

